# Mental fatigue impairs endurance performance in a time-to-exhaustion handgrip task: psychophysiological markers of effort engagement dynamics

**DOI:** 10.3389/fpsyg.2025.1611135

**Published:** 2025-07-23

**Authors:** Sarvenaz Daneshgar-Pironneau, Michel Audiffren, Abdelrahni Benraïss, Angèle Métais, Nathalie André

**Affiliations:** ^1^Centre de Recherches sur la Cognition et l’Apprentissage, Université de Poitiers, CNRS, Poitiers, France; ^2^Laboratoire Motricité, Interactions, Performance, Nantes Université, Nantes, France

**Keywords:** electroencephalography, executive function, heart rate variability, mid-frontal theta, self-control, Stroop test

## Abstract

**Introduction:**

A growing body of literature showed that mental fatigue induced by an effortful task leads to an impairment in a subsequent physical performance. The principal aim of this experimental study was to reproduce the effect of mental fatigue on endurance performance while investigating the effort deployment in the fatiguing and control tasks that precede the physical task.

**Methods:**

Participants performed the following task sequence in a between-subjects design (*N* = 16 in each group): a time-to-exhaustion handgrip task at 13% of maximal voluntary contraction, a 30-min mental task (Stroop task or documentary watching task) and the handgrip task again. Psychophysiological data were recorded on a continuous basis during the whole experiment.

**Results:**

Mental fatigue was induced successfully: behavioral and psychophysiological data suggest gradual disengagement of effort in the fatiguing task but not in the control task (increased reaction time and error rate as the function of time in the Stroop task; higher mid-frontal theta during the Stroop task compared to the control task; decreased stimulus-locked theta rhythm over time during the Stroop task; increased low frequency heart rate variability during the Stroop task). In addition, performance decrement in the time to exhaustion handgrip task was larger after the Stroop task than after the documentary viewing task (*d =* 0.818).

**Conclusion:**

This study highlights the importance of assessing both performance and effort engagement to understand mental fatigue. Despite signs of effort disengagement during the Stroop task, mental fatigue still impaired the subsequent physical performance.

## 1 Introduction

In many professions, workers perform effortful tasks for long periods of time during a working day. This leads to a state of acute mental fatigue associated with an increased risk of accidents, a decline in the quality of the work output or higher error rate in the decisions made (Swaen et al., 2003; Danziger et al., 2011; Jia et al., 2022; Zhang et al., 2023; Aria et al., 2024). Acute mental fatigue has also been identified as an important limiting factor of physical performance (Marcora et al., 2009; Van Cutsem et al., 2017a, 2017b; Brown et al., 2020).

Acute mental fatigue is defined as a change in the psychophysiological state of an individual during and/or following prolonged periods of effortful cognitive activity (Marcora et al., 2009). Manifestations of mental fatigue include a decreased motivation to exert effort (Inzlicht et al., 2014), increased feelings of “tiredness” and “lack of energy” (Boksem and Tops, 2008), decreased endurance performances (Giboin and Wolff, 2019), increased choice impulsivity towards immediate rewards (Blain et al., 2016) and increased difficulty to control automatic behaviors such as eyeblinks (Wascher et al., 2014a).

However, although the subjective and behavioral manifestations of acute mental fatigue are now well documented, there remains a pressing need for reliable neurobiological or psychophysiological markers to track its development over time. The objective of this study is to contribute to the advancement of knowledge in this field by exploring the variations of several psychophysiological indices during the fatigue-inducing task.

The most widely used protocol for investigating mental fatigue, known as the sequential-task protocol, was introduced by Muraven et al. (1998) to study failures in self-control (i.e., ego depletion) in social psychology research. It was then used extensively in sports science to study the effects of mental fatigue on physical performance (Giboin and Wolff, 2019). In this protocol, participants successively perform two tasks under two different conditions. The first task can be either highly effortful (fatiguing task) or weakly effortful (control task). This first task is followed by a second effortful physical task (dependent task), which is identical in the two conditions described above. If the fatiguing task really does induce mental fatigue, and the control task requires little mental effort, performance on the dependent task must be worse after the fatiguing task. Mental fatigue can also be observed during the fatiguing task as a function of the time on task (e.g., Mangin et al., 2022). This effect is known as the “time-on-task effect,” and corresponds to a progressive deterioration in performance over the time spent on the fatiguing task.

Ego depletion and acute mental fatigue share similarities but are distinct phenomena. In experiments from social psychology, ego depletion is generally induced by short tasks (i.e., 8 min in average) that require self-control, i.e., the ability to resolve a motivational conflict (Forestier et al., 2022), whereas in experiments from sport sciences mental fatigue is induced by long (> 30 min) and effortful tasks that do not necessarily require self-control (e.g., working memory tasks). The duration and the cognitive load of the fatiguing task are two crucial parameters in the effort-based theoretical frameworks supporting mental fatigue (e.g., André et al., 2019; Pessiglione et al., 2025) whereas it was not really considered in the initial version of the strength model of self-control (Baumeister et al., 2007; Baumeister and Vohs, 2016).

The meta-analysis of Giboin and Wolff (2019) showed that the type of the dependent physical task moderates the size of the effect of mental fatigue. More specifically, isolation tasks (e.g., handgrip tasks) seem to be more sensitive to mental fatigue compared to whole-body endurance tasks (e.g., running or cycling tasks). The authors of the meta-analysis hypothesized that the higher sensitivity of isolation tasks could be due to a higher attentional control demand in this type of endurance task compared to whole-body endurance tasks. For that reason, time-to-exhaustion (TTE) isolation handgrip tasks seem to be a good choice as dependent tasks to observe mental fatigue effects. In addition, the TTE handgrip task has been shown to be a reliable indicator of fatigue resistance (Bautmans and Mets, 2005; De Dobbeleer et al., 2017). Prior research has also found that performing isometric handgrip exercises to the point of exhaustion can impair subsequent cognitive performance, such as in a Stroop task (Brown and Bray, 2015).

In effort-based models of acute mental fatigue (e.g., André et al., 2019; Pessiglione et al., 2025), effort is viewed as a multifaceted concept. First, it is viewed as a decision-making mechanism anchored in the anterior cingulate cortex (ACC) that balances costs and benefits associated with the achievement of the task goal and allocates/mobilizes available resources (i.e., energy and cognitive control) to reach the task goal if and only if benefits outweigh costs. In this framework, mental fatigue is viewed as a cost that grows over time throughout an effortful task and leads to a reduction in the capacity and willingness to deploy energy and cognitive control. Mental effort is also viewed as a control signal, a product of the mechanism of effort, that is generated when resources need to be deployed to reach a goal. Theta oscillations generated by the ACC would reflect this control signal associated with effortful control (Holroyd, 2016; Holroyd and Umemoto, 2016; Vassena et al., 2017).

The choice of an adequate cognitive fatiguing task is crucial to induce an acute mental fatigue effect in the following dependent task. All theories of mental fatigue (e.g., Hockey, 1997; André et al., 2019; Pessiglione et al., 2025) posit that the fatiguing task needs to be cognitively demanding (i.e., a high cost in cognitive control). Tasks requiring executive functions, such as inhibitory control, updating of working memory and/or cognitive flexibility, are good candidates for that purpose (Miyake et al., 2000; Diamond, 2013). Two positive large-scale neuronal networks are systematically activated when someone performs a task requiring executive functions (Seeley et al., 2007; Menon and Uddin, 2010; Seeley, 2019; Menon, 2023). The first is the central executive network, anchored in the dorsolateral prefrontal cortex (DLPFC) and the posterior parietal cortex (PPC), which are involved in executive functioning and cognitive control. The second is the salience network, anchored on the anterior cingulate cortex (ACC) and the ventral anterior insula, which are implicated in effort-based decision-making, allocation of resources to achieve goals, and the generation of effort-related signals. A modified Stroop task was chosen to elicit mental fatigue for the main following reasons: (1) it involves inhibitory control and cognitive flexibility, two core executive functions; (2) DLPFC and ACC are clearly activated during the Stroop task (e.g., Carter et al., 1995; Mitchell, 2005).

Mental fatigue manifests differently depending on the strategy adopted by the individual (Hockey, 1997, 2011): either a disengagement from mental effort over time accompanied by a decline in performance, or an increase in mental effort accompanied by maintained performance. Therefore, the objective diagnosis of acute mental fatigue requires the simultaneous measurement of two dependent variables: performance and mental effort. However, in the large majority of the studies inducing mental fatigue with the sequential-task protocol, the level of effort deployment throughout the cognitive tasks (i.e., the fatiguing and control tasks) is rarely examined. Lorcery et al. (2024) used the pre-ejection period (i.e., an index of sympathetic activity) to examine the deployment of effort during a 30-min modified fatiguing Stroop task. They observed a decrease in sympathetic activity throughout the cognitive task; results that suggest a progressive disengagement of effort or a habituation process. However, sympathetic activity often used as a concomitant of effort engagement (i.e., mobilization of energy) presents two caveats. First, as mentioned just above, the activity of the autonomic nervous system, which is composed of the sympathetic nervous system and the parasympathetic nervous system, is sensitive to habituation and could be inappropriate to assess effort mobilization over time in long cognitive tasks. Second, the activity of the sympathetic system is not under the direct control of the ACC and is consequently a distal marker of the control signal generated by the ACC. Brain activity markers such as prefrontal midline theta oscillations recorded with the use of electroencephalography (EEG) are clearly associated with cognitive control and sustained mental effort (Cavanagh and Frank, 2014; Holroyd and Umemoto, 2016). In addition, numerous studies support the view that ACC is an important source of theta oscillations and that prefrontal midline theta spectral density is a good index of ACC output (Kao et al., 2013; Sauseng et al., 2007; Holroyd and Umemoto, 2016; Tran et al., 2020). Moreover, EEG studies have shown an increase in theta power density during tasks requiring mental effort (Smit et al., 2005). We can infer from the above that mid-frontal theta spectral density could be a good marker of mental effort during the fatiguing and the control tasks of the sequential-task protocol.

However, apparent contradictory results were reported concerning EEG activity recorded during sustained attentional tasks. Numerous researchers observed an increase in overall theta rhythm over time that was generally interpreted as an increase in mental fatigue with time on task (e.g., Van Cutsem et al., 2017a, 2017b; for reviews see Borghini et al., 2014; Tran et al., 2020), whereas others found a decrease in amplitude of event-related potentials or in theta rhythm that was interpreted as a decrease in mental effort with time on task (e.g., Boksem et al., 2006; Wascher et al., 2014b). More recently, Arnau et al. (2021) reconciled these contradictory findings showing that spectral power in the theta band increased with increasing time-on-task during the intertrial interval (non-task-related theta rhythm) whereas it decreased over the course of the experiment in response to the imperative stimulus of the cognitive task (stimulus-locked task-related theta rhythm). To achieve the objective to examine the variations of mental effort during the fatiguing task, we chose to focus on stimulus-locked task-related theta wave density rather than overall theta rhythm.

In addition to mid-frontal theta assessed by EEG, other psychophysiological indices, such as cardiac reactivity, can also be considered as indices of effort engagement (Obrist, 1981). Heart rate variability (HRV), or the analysis of beat-to-beat intervals, is one of the most common and reliable physiological measurements used to assess mental effort (Mukherjee et al., 2011). The sympathetic and parasympathetic nervous systems influence HRV, which can vary depending on the subject’s physiological and psychological state (Ernst, 2017). During the performance of a task that requires mental effort, the previous literature has mainly reported a decrease in heart rate variability (Capa et al., 2008). Three heart-rate variability indices are frequently reported in the literature: one time-domain index, the SDNN (standard deviation of NN intervals), and two frequency-domain indices, the LF power (log power of the low-frequency band from 0.04 to 0.15 Hz) and HF (log power of the high-frequency band from 0.15 to 0.4 Hz). The SDNN demonstrates the components that are responsible for the variability in the recording period (Malik, 1996). High frequency can be considered the cardiac parasympathetic tone index (Reyes del Paso et al., 2013) since it reflects vagal tone (Malik, 1996; Laborde et al., 2017). Low frequencies are assumed to be markers of cardiac outflow, which is under the influence of both the sympathetic and parasympathetic autonomic nervous systems (Malik, 1996; Laborde et al., 2017). A recent systematic review showed that HRV is a reliable autonomic marker for time-on-task (TOT) induced fatigue as expected in the present experiment (Csathó et al., 2024). Low frequency index and time domain indices of HRV, such as the SDNN, tend to increase with time-on-task reflecting an increase in parasympathetic activity and suggesting that when participants work on a cognitively demanding task for a prolonged period, they may be likely to be disengaged from the task (e.g., Van Cutsem et al., 2022).

Consequently, the electrophysiological changes in effort engagement throughout the fatiguing task and the control task of the sequential-task protocol will be assessed through mid-frontal theta density and heart rate variability indices. Based on all the above theoretical framework, we first hypothesized that the participants would show a worse endurance performance in the TTE handgrip task after the fatiguing task compared to after the control task. We also hypothesized higher mid-frontal theta density and lower HRV during the fatiguing task than during the control task (i.e., more effort engagement in the fatiguing task). Finally, we hypothesized a progressive decrease in mid-frontal theta power and an increase in HRV (i.e., increase in parasympathetic activity and decrease in sympathetic activity) as a function of TOT during the fatiguing task (i.e., progressive disengagement of effort).

## 2 Materials and methods

### 2.1 Participants

The present experiment was a pilot study and we did not determine the sample size *a priori*. However, a *post hoc* computation of the achieved power was conducted and presented in the results and discussion section. Thirty-two undergraduate students in sport sciences at the University of Poitiers were recruited to participate in this study. They were randomly assigned into two groups: a control group and an experimental group. The characteristics of the participants can be found in Table 1. All of the participants were native French speakers and right-handed except one in the experimental group who was left-handed. Inclusion criteria are described in Supplementary Material S1.

**Table 1:** Participant’s characteristics.

Variable	Experimental group	Control group	*p* value
Age (years)	21.73 (2.14)	21.25 (1.61)	0.481
Sex (M/F)	8/8	7/9	0.723
BMI (kg/m^2^)	23.96 (2.69)	21.50 (2.01)	0.006*
Trait self-control (13–65)	47.75 (6.19)	44.56 (4.56)	0.108
MVC (kg)	18.68 (6.79)	17.00 (3.56)	0.390
TTE-1 (min)	8.32 (4.30)	5.31 (1.53)	0.012*

* = *p* value <0.05; M = Male; F = Female; BMI = Body mass index; MVC = Maximal voluntary contraction; TTE = Time to exhaustion. The means and standard deviations (in brackets) are reported for each group in the two central columns.

The experimental procedure was explained to the participants via a written information sheet. Then, they signed a consent form to indicate their agreement. Finally, the participants received course credit in exchange for their participation. This experiment was conducted in accordance with the Declaration of Helsinki and with the approval of the University of Tours-Poitiers ethics committee (serial number CER-TP 2019-01-02).

### 2.2 Procedures

**Figure 1:**
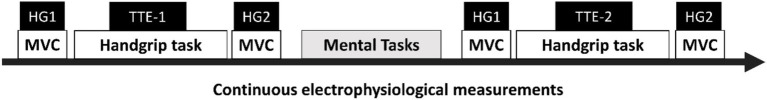
Time course of the sequential-task protocol. Cognitive task: experimental group = modified incongruent Stroop task; control group = documentary viewing task. MVC: maximal voluntary contraction. Handgrip task: time-to-exhaustion (TTE) task. HG1: measurement of MVC before the TTE task. HG2: measurement of MVC after the TTE task. TTE-1: TTE handgrip task performed before the mental task. TTE-2: TTE handgrip task performed after the mental task.

This experiment occurred during a single session and the participants in both groups underwent the exact same procedures. The session started with participants completing different questionnaires and tests to verify the inclusion criteria (see Supplementary Material S2 for more details). Then, the participants familiarized themselves with the mental and physical tasks. Afterwards, they were equipped with EEG and electrocardiography (ECG) electrodes. Finally, they underwent the sequential stages of the experimental protocol as shown in Figure 1 while their electrophysiological data were recorded on a continuous basis.

First, the participants were asked to perform a TTE handgrip task to determine their physical performance baseline, while their maximal voluntary contraction (MVC) was assessed before and after this task. Second, they performed a 30-min mental task that differed in each of the two groups. The experimental group performed a 30-min modified incongruent Stroop task tapping two executive functions: inhibitory control and cognitive flexibility. The control group watched a 30-min documentary video. After the completion of the mental task, the participants repeated the TTE handgrip task and the MVC measurements before and after this physical task. Several other subjective measurements were made throughout the session at four different times: T1 = Baseline, T2 = Post-Handgrip task 1/ Pre-Mental Task, T3 = Post-Mental Task/Pre-Handgrip task 2, T4 = Post-Handgrip Task. We used a computerized visual analog scale (VAS) with an adapted scale (0 to 100%) to rate the level of their perceived fatigue (T1 to T4), the motivation to perform the physical task (T1 and T3), the perceived difficulty of the mental task (T3) and finally the level of boredom experienced during the mental tasks (T3). The motivation to perform the dependent task and subjective mental fatigue were already assessed with a similar VAS (Brown and Bray, 2017), and this type of VAS was validated by Wewers and Lowe (1990). Finally, a computerized version of the Brief Self-Control Scale (Tangney et al., 2004) was used to assess the participants’ trait self-control due to the probable influence of this trait on mental fatigue. Previously, it has been argued that individuals with a high self-control trait tend to demonstrate lower mental fatigue effects than individuals with a low self-control trait (DeWall et al., 2007).

### 2.3 Tasks

#### 2.3.1 Time-to-exhaustion (TTE) task

The dynamometer (TSD121C, BIOPAC), the AcqKnowledge software, version 4.2 (BIOPAC Systems Inc., Goleta, CA, United States) and the MP160WSW data acquisition unit were used to record the force signal in the handgrip tasks. Data were recorded online at a sampling rate of 2000 Hz and later stored and analyzed offline.

**Figure 2:**
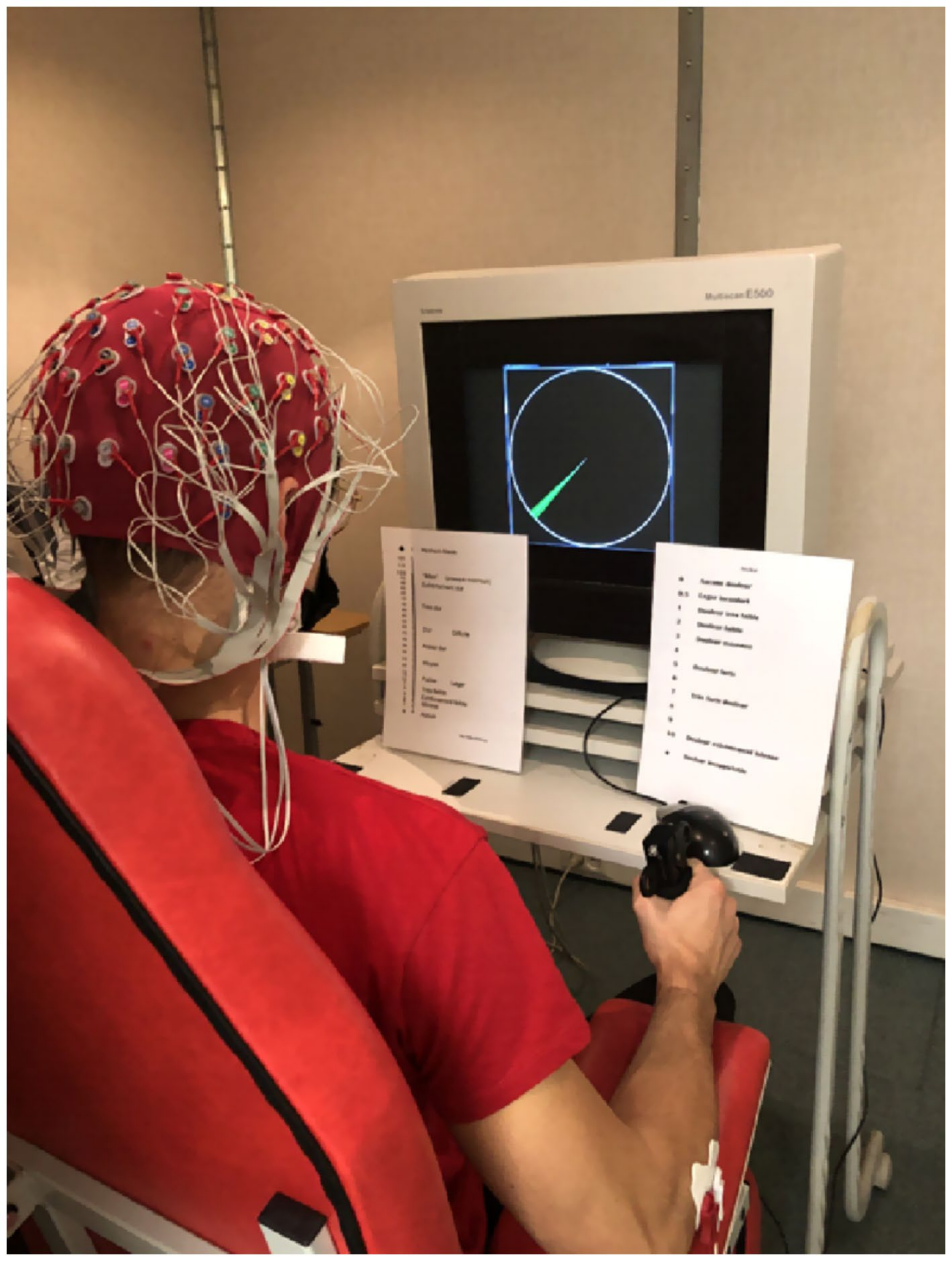
Overview of the experimental setup. The participants equipped with a 64-channel Biosemi EEG headset while performing the handgrip task. The maximized version of the force gauge is displayed on the screen of the computer. The green arc defines the target zone (i.e., a range between 12 and 14% of MVC), which was visible throughout the duration of the task. The participants had to maintain the gauge needle in this green area until exhaustion. On the left side of the screen, the CR100 effort scale. On the right side of the screen, the pain scale.

To perform the handgrip tasks, the participants were asked to sit on a fixed chair with an arm support allowing for the imposition of an angle of ~90 degrees with their elbow and forearm (see Figure 2). The participants were also asked to remain in this anatomically neutral position throughout the handgrip task.

The participants started the physical task by performing a maximal voluntary contraction (MVC) while squeezing a hand dynamometer as strongly as possible for a duration of 3 s. This process was repeated until the time that the participants could not score higher in contraction peak force compared to their previous performances. Between each MVC measurement, there was a 30-s rest. The highest MVC measured before the first TTE handgrip task was used throughout the session as the reference to calculate the targeted force (i.e., 13% MVC) in the TTE handgrip task.

The MVC was used to calibrate the target zone for the TTE handgrip task (see the gauge on Figure 2). The target zone area was defined as a green arc representing 12 to 14% of the MVC. The force exerted by the participant was indicated by the needle of a gauge. The perimeter of the gauge represented 100% of the participant’s MVC. The participants were asked to maintain the needle within the range of the green arc until exhaustion. TTE was evaluated offline and considered the duration of the isometric contraction from the onset of the force signal (i.e., as soon as the participant’s force reached 12% MVC) to the exhaustion time (i.e., as soon as the participant stays below 12% MVC for more than 2 s).

During the TTE handgrip task, the participants had to squeeze the hand dynamometer and stay between 12 to 14% of the MVC until exhaustion (see Supplementary Material S4 for more details). This TTE handgrip task was used as the dependent task. During the TTE task, every 30 s, we asked the participants to rate their perception of effort and their perception of pain. After the TTE handgrip task, the participants repeated the procedure to measure the MVC. This cycle of handgrip tasks (i.e., CMV measurement, TTE task, CMV measurement) was repeated after the mental task (see Figure 1).

A percentage of 13% of MVC was chosen to increase the duration of the task and enhance the endurance component of the task. This intensity was selected among a large sample of different intensities during a pilot study in order to produce, in average, maximal durations of squeezing that are neither too short (< 3 min) nor too long (> 20 min). This low-intensity TTE handgrip task exhibited good reliability with regard to a set of data collected in a previous unpublished study (*n* = 55; ICC3 [A, 2] = 0.868; 95% CI = [0.774, 0.923]).

To assess the extent of muscle fatigue and to ensure that the participants performed the handgrip task as much as they could, we compared the difference between the MVC peak force measured before and after the TTE handgrip task (see Figure 1). Because we used a between-subjects design, we measured MVC and TTE before the mental task to be sure that both groups were comparable on these two dependent variables.

Perception of effort and perception of muscle pain were measured throughout the TTE handgrip task (see the scales below the screen on Figure 2). The perception of effort is referred to as the effort intensity necessary to squeeze the dynamometer while breathing and remaining in the green zone (Mangin et al., 2021) on the CR100 scale (Borg and Kaijser, 2006). The perception of muscle pain is defined as the perceived pain intensity in their forearm muscles during the TTE handgrip task (Mangin et al., 2021) on the Cook scale (O’Connor and Cook, 2001).

The *individual isotime method* (Nicolo et al., 2019) was used to analyze the participants’ perceptions of muscle pain and effort. This method considers the shortest performance record of the participant on the handgrip task as his or her 100% individual isotime. Subsequently, the corresponding isotime points to 0, 33, and 66% of the individuals were calculated for their shortest performance. According to the calculated isotime points, their level of muscle pain and effort was assessed as a function of TOT.

#### 2.3.2 Incongruent Stroop task

We used a computerized modified Stroop task designed with the E-prime software, version 2.0 (Psychological Software Tools, Pittsburgh, PA, United States), as a fatiguing task for the experimental group. The participants sat in front of a screen and responded orally to the visual stimuli that appeared at the middle of the screen. Participants’ response was recorded with an S-R response box.

The participants underwent 888 trials of 2 s each. Fifty percent of the trials were “reading” trials (i.e., the participants have to read the word) and the other 50% were “naming ink color” trials (i.e., the participants have to name the color of the ink). These two types of trials were presented in a randomized order and primed with a preparatory signal.

Every single trial of the Stroop task started with a fixation point in the form of a cross that lasted 400 ms. The fixation point could be enclosed in a circle or a square for a duration of 50 ms, and then the cross remained on the screen alone. Immediately thereafter, a color name (green, red, yellow or blue) written in another ink color (e.g., “yellow” written in red) was displayed on the screen. Participants were instructed to read the word when a square appeared (“reading” trials) and to name the color of the ink when a circle appeared (“naming ink color” trials). The color word lasted on the screen until the participant’s response. If the participants did not answer (omission) or had a reaction time longer than 1,250 ms, the color word lasted 1,250 ms and was followed by a fixation cross lasting 300 ms. When the participant answered before 1,250 ms had elapsed, the fixation cross remained in the middle of the screen for the same amount of time (1,250 ms + 300 ms) to have a duration of 2 s for each trial. The verbal responses of the participants were recorded via two microphones, one to record the word spoken by the participant (headset) and the other to measure his or her response time (fixed microphone).

The presentation order of the two categories of trials was completely random. The block of trials included 50% trials in which the participants had to read the color word and 50% of trials in which they had to name the font color for the sake of increasing the task difficulty by engaging cognitive flexibility and inhibitory control (Mangin et al., 2021) and limiting the learning effect (Dulaney and Rogers, 1994).

Performance in the modified Stroop task was analyzed as a function of time-on-task and type of trial. Data were divided into 4 consecutive periods of 222 trials lasting 7 min and 30 s. For each time period and type of trial, we calculated the mean reaction time for correct responses (RT) and error rate.

#### 2.3.3 Documentary watching task

We chose a documentary movie named Earth (Fothergill and Linfield, 2009) as a control task for the control group. This documentary was tested as emotionally neutral and weakly boring (Mangin et al., 2021). Later, the participants were asked to complete a multiple-choice questionnaire related to the content of the documentary to verify whether they were actively watching the movie or not.

### 2.4 Psychophysiological measures

#### 2.4.1 EEG acquisition and analyses

The EEG data were collected from a 64-channel Biosemi EEG headset with the electrode distribution based on the international 10–20 system. For the sake of better eye movement artifact detection, the participants’ ocular activities were recorded via three electrooculogram (EOG) electrodes (two on the outer canthus of each eye and one on the infraorbital region of the right eye). The EEG and EOG signals were continuously recorded online throughout the whole experiment using the ActiView Biosemi version 6.05 acquisition system at a frequency of 2000 Hz referenced online to the average of the right and left mastoids.

The MATLAB R2020b programming platform (MathWorks Inc., Natick, MA, United States), together with the open source EEGLAB 2021.0 toolbox (Delorme and Makeig, 2004), was used for offline data analysis. Data preprocessing steps and artifact rejection methods were adopted from a very recent reproducible workflow by Pernet et al. (2021). First, data were down-sampled to 250 Hz, and a basic low-pass FIR filter to the higher edge of 40 Hz was applied to avoid 50-Hz line noise. Then, the clean_rawdata plugin in EEGLAB (version 2.2) was used to high-pass filter the data at 0.5 Hz (transition band [0.25 0.75]), as well as remove the bad channels (any channel with at least a 5-s flat line and/or with less than 0.8 robust estimate correlation to the other channels were considered bad channels). Next, the data were re-referenced to the average of the existing channels. Afterward, the data were decomposed into independent components using the ICA algorithm (runica algorithm with rank reduction based on the number of channels = −1, considering the average reference). An automatic algorithm was applied to label the components using ICLabel (Pion-Tonachini et al., 2019), and components labeled as muscle activities and eye movements with greater than 80% probability were omitted from the data. Then, the residual artifacts were removed using the artifact subspace reconstruction (ASR) algorithm parametrized to a 20-burst detection criteria threshold (Chang et al., 2018, 2020). Finally, we inspected the data visually and rejected any nonbrain components or artifactual portions of the data that were not detected hitherto by the automatic algorithm. No more than half of the components in each data point were rejected (the maximum number of component rejections was 32 out of 64). A power spectral analysis was applied to observe the differences in the overall of theta wavebands (4–7 Hz) during the Stroop task performance as opposed to the video task. The “spectopo” function in EEGLAB was used to perform the power spectral analysis and to compute each component power spectrum using the fast Fourier transform (FFT) with the following parameters: a window size of 1 s with a 50% overlap. We were also interested in the effect of time-on-task during the Stroop task on the task-related theta waveband power; therefore, we also studied the data by dividing the 30 min corresponding to the Stroop task into 4 consecutive task periods of 7.5 min. Segmentation was performed after the initial preprocessing steps to ensure the use of clean and reliable data with minimized influence of noise and artifacts on the segmented data and further analysis.

According to the methodology used by Arnau et al. (2021), we conducted an analysis of stimulus-locked theta power during the Stroop task. Throughout the Stroop task, when the color word was displayed on the screen, a marker was sent to ActiView Biosemi with the help of the Eprime program indicating that the ongoing trial is either a ‘naming ink color’ trial or a ‘reading’ trial. Later, during the EEG offline analysis, the data were analyzed in the following time window: from 100 ms before the stimulus onset to 2000 ms after this onset. After removing the baseline recorded during the time window from 100 ms before the stimulus to the onset of the stimulus, we separated the 444 trials belonging to the ‘naming ink color’ condition from the 444 trials belonging to the “reading” condition for further analyses. Then, the power spectral analysis was applied to observe the changes in theta wave band by comparing these two types of trial (reading vs. naming ink color) as the function of TOT.

Concerning the source localization technique, the DIPFIT plugin (Oostenveld and Oostendorp, 2002) in the EEGLAB 2021.0 toolbox (Delorme and Makeig, 2004) was used to calculate an equivalent current dipole model for each independent component through a four-shell spherical head model. A bilaterally symmetric dual dipole model was fitted for the components with bilaterally distributed scalp maps. Only components located inside the model of brain volume, for which their best-fitting single or dual equivalent dipole showed less than 15% residual variance from the spherical forward-model scalp projection, were contemplated for further analysis.

#### 2.4.2 Heart rate variability

Heart rate variability (HRV) was continuously recorded using the Electrocardiograph (BIOPAC Systems Inc., Goleta, United States) and AcqKnowledge 4.2 software (BIOPAC Systems Inc., Goleta, CA, USA) at a frequency of 2000 Hz by placing three EL503 electrodes on the participant’s thorax as recommended by the American Heart Association (Kligfield et al., 2007). The Kubios HRV Premium software, version 3.5.0 (Tarvainen et al., 2014), was used to analyze the data. After applying the medium automatic artifact correction algorithm available in Kubios, the residual artifacts were rejected via manual inspection. One subject in the control group was excluded from further analysis due to the low quality of his or her ECG data. We studied the changes in HRV on the temporal basis of four equal time windows of 7 min and 30 s. We analyzed the data by examining the time and frequency domain parameters of HRV.

### 2.5 Statistical analysis

The statistical analyses were performed with Jamovi software, version 2.2.5, Jasp software, version 0.16.3.0, and Statistica, version 14.0.0.15. The statistical analysis of EEG data was performed in EEGLAB using EEGLAB Study parametric statistics. We set the alpha level for statistical significance to *α* = 0.05. When the results were significant and marginal, the effect sizes were calculated: Cohen’s d for the t test (only Student’s t test was used in this study), rank biserial correlation for its equivalent nonparametric Mann–Whitney U test and partial eta square (*η_p_^2^*) for analysis of variance (ANOVA). When testing an effect involving a repeated-measures factor with more than two levels (e.g., time-on-task), we applied a Greenhouse–Geisser correction to consider any violation of the sphericity assumption. For EEG data, the FDR multiple comparison correction available in EEGLAB statistics was considered.

## 3 Results

Principal characteristics of the two groups of participants were compared to check whether they were homogenous or not (see Table 1). ANOVA with Group (experimental vs. control) as the between-subjects factor was performed on age, body mass index (BMI), trait self-control, MVC, and average TTE on the handgrip task performed at the beginning of the session (TTE-1). Table 1 shows that the two groups significantly differed in BMI and TTE-1 only. The control group had significantly lower BMI and TTE-1 than the experimental group. Consequently, BMI was used as a covariate in ANOVA leading to a significant effect and including the factor Group as a between-subjects factor. In the same way, the difference between TTE-1 and TTE-2 was used as the main outcome to test the effect of mental fatigue, instead of TTE-2 only. The chi-square statistic on the percentage of men and women showed no significant difference between the groups.

### 3.1 Manipulation check

#### 3.1.1 Task difficulty

The results of the two-sample t test demonstrated that the participants in the experimental group perceived the Stroop task (*M* = 74.9, *SD* = 19.7) to be more difficult than the control group did for the documentary viewing task (*M* = 20.1, *SD* = 22.2): *t* (30) = −7.39, *p* < 0.001, *d* = −2.61.

#### 3.1.2 Subjective feeling of fatigue

**Figure 3:**
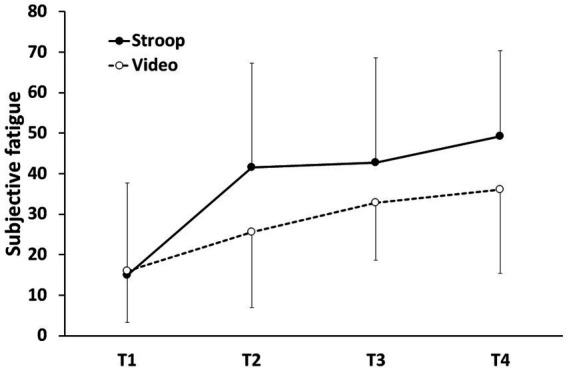
Subjective fatigue as a function of session time. T1 = baseline, T2 = post-handgrip task 1/pre-mental task, T3 = post-mental task/pre-handgrip task 2, T4 = post-handgrip task 2. Errors bars represent standard deviations.

An ANOVA with Group (Experimental vs. Control) as the between-subjects factor and Time of measurement (T1 = Baseline, T2 = Pre-Mental Task, T3 = Post-Mental Task, T4 = Post-Handgrip Task) as the within-subjects factor was performed on the subjective feeling of fatigue. We applied a Greenhouse–Geisser correction to consider any violation of the sphericity assumption. The results showed that the interaction Group x Time of measurement and the main effect of Group did not reach significance. In contrast, the effect of Time of measurement reached significance: *F* (2.774, 83.212) = 22.299, *p* < 0.001, *η^2^_p_* = 0.426. According to a planned comparison conducted to explain the effect of time of measurement, we observed a significant, linear increase in fatigue level as time progressed, regardless of the first cognitive task: *t* (90) = 7.637, *p* < 0.001. These results suggest that although participants perceived the Stroop task as more difficult than watching the documentary, their fatigue level increased comparably in both groups from the beginning to the end of the session (see Figure 3).

#### 3.1.3 Boredom

The two-sample t test was performed to compare perceived boredom during the fatiguing versus control tasks. The effect of Group (experimental vs. control) did not reach significance: *t* (39) = − 1.11, *p* = 0.274. Unexpectedly, the Stroop task performed by the experimental group (*M* = 54.69; *SD* = 27.58) was not perceived as more boring than the documentary viewing task (*M* = 45.13; *SD* = 20.41) performed by the control group. The level of boredom was moderate in both tasks.

#### 3.1.4 Motivation to perform the TTE handgrip task

Repeated-measures ANOVA with Group (Experimental vs. Control) as a between-subjects factor and Time of measurement (T1 = Baseline/ Pre-Handgrip task 1, T2 = Post-Mental Task/Pre-Handgrip task 2) as a within-subjects factor was conducted on the motivation to perform the TTE handgrip task. According to the results, the interactions Group x Time, and the main effects of Time of measurement and Group did not reach significance: *F* (1, 30) = 0.030, *p* = 0.862, *η^2^_p_* = 0.001; *F* (2.57, 77.12) = 3.216, *p* = 0.083, *η^2^_p_* = 0.097; and *F* (1, 30) = 2.14, *p* = 0.154, *η^2^_p_* = 0.067, respectively. The level of motivation was quite high in both groups (*M* = 69.55; *SD* = 28.11).

#### 3.1.5 Maximal voluntary contraction

ANOVA with Moment (before the mental task vs. after the mental task) and Repetition (HG1 vs. HG2) as within-subjects factors and Group (experimental vs. control) as a between-subjects factor was performed on MVC performance. None of the interactions reached significance. The simple main effect of Moment was significant: *F* (1, 30) = 83.254, *p* < 0.001, *η^2^_p_* = 0.735. The mean value of MVC was significantly lower after the TTE handgrip task (*M* = 13.3 kg, *SE* = 0.738) than before it (*M* = 17.5 kg, *SE* = 0.862). In addition, we observed a significant effect of Repetition: *F* (1, 30) = 4.948, *p* = 0.034, *η^2^_p_* = 0.142. The mean value of MVC was significantly higher during the first handgrip task (*M* = 15.7 kg, *SE* = 0.826) compared to the second handgrip task (*M* = 15.0 kg, *SE* = 0.745). On average, the participants’ MVC was reduced by 23.33% after the TTE handgrip task. This decrease in MVC reflects a lower capacity to produce a muscular force. The effect of Group did not reach significance: *F* (1, 30) = 0.321, *p* = 0.575, *η^2^_p_* = 0.011.

#### 3.1.6 Performance in the Stroop task

**Figure 4:**
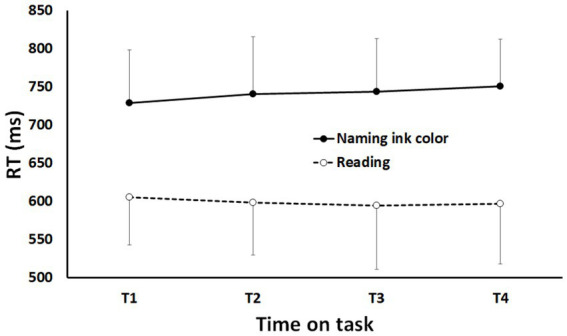
Mean reaction time as a function of time-on-task (T1-T4) and type of trials (reading vs. naming ink color). Error bars represent standard deviations.

To assess the performance in the Stroop task, two separate ANOVAs with Time-on-task (TOT; T1, T2, T3 & T4) and Type of trial (reading vs. naming ink color) as within-subjects factors were conducted on the mean reaction time (RT) and error rate. The interaction Time-on-task x Type of trial reached significance for mean RT (see Figure 4): *F* (2.282, 34.227) = 4.881; *p* < 0.05; *η^2^_p_* = 0.245. This interaction is explained by the difference between “reading” trials and “naming ink color” trials: RT increased with time-on-task for ‘naming ink color’ trials but remain stable for ‘reading’ trials (see Figure 4). The interaction Time-on-task x Type of trial and the simple effect of Time-on-task did not reach significance for error rate.

In addition, the simple effect of Type of trial is significant for mean RT and error rate: *F* (1, 15) = 155.904; *p* < 0.0001; *η^2^_p_* = 0.912 and *F* (1, 15) = 156.565; *p* < 0.0001; *η^2^_p_* = 0.913, respectively. As expected, ‘reading’ trials led to shorter mean RT (629.44 ms vs. 741.19 ms) and smaller error rate (6.66% vs. 15.42%) than ‘naming ink color’ trials. Results concerning the documentary viewing task are presented in Supplementary Material S3.

#### 3.1.7 EEG activity during the mental tasks

**Figure 5:**
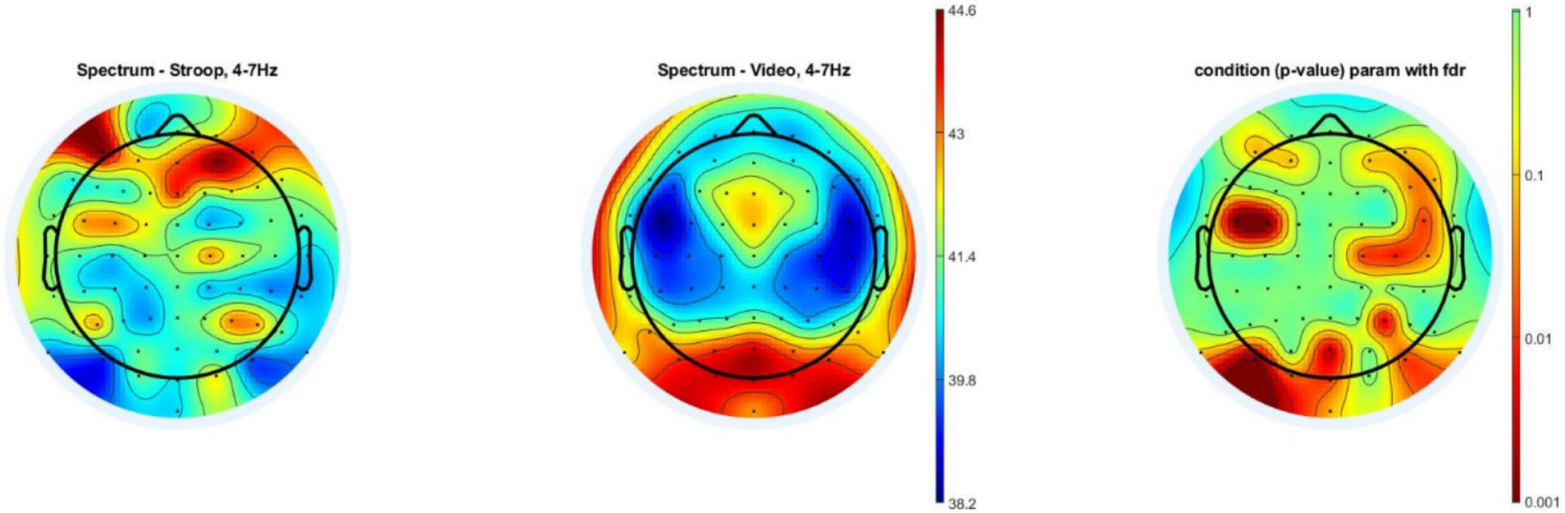
Power spectral density analysis during the mental tasks. The left topographic color plot shows the mean theta power spectral density for all 16 subjects in the experimental group during the 30-min Stroop task. The central topographic color plot shows the mean theta power spectral density for all 16 subjects in the control group during the 30-min documentary viewing task (Video). The right topographic color plot shows the *p* values found when using the unpaired t test with FDR multiple comparison correction to compare the theta power differences between the two groups. The color bar scale located in the middle of the figure shows the log power 10 (μV^2^). The greater that the color tends toward dark red, the higher that the density of the theta waves is. The second color bar scale on the right shows the *p* value. The greater that the color tends toward dark red, the greater that the *p* value tends toward 0.001.

We used the unpaired t test with FDR correction for multiple comparisons to compare the overall theta wave band power spectral density during the Stroop task compared to that of documentary viewing task. A statistically significant higher theta power spectral density was observed mainly in the frontal, prefrontal and central areas during the Stroop task compared to the documentary viewing task, more specifically in the AF7, AF3, FC5, FC3, AF4, AF8, F8, F6, FC6, C6, C4, C2 and P4 electrodes. However, we also detected significantly higher theta power during the documentary viewing task, mainly in occipital areas, more precisely the POz, O1, Iz, Oz, PO7 and PO8 electrodes (see Figure 5). In addition, we conducted a repeated-measures MANOVA on overall theta spectral density only in the region of interest (R) above the dorsolateral prefrontal cortex (DLFC), as in the study of Van Cutsem et al. (2017a, 2017b), with Electrodes (F3, Fz and F4) as a within-subjects factor and Group (Stroop, Video) as a between-subjects factor. The effect of Group and the interaction Group x Electrode did not reach significance: *F* (1, 30) = 0.41, *p* = 0.527 and *F* (2, 29) = 2.167, *p* = 0.133, respectively.

**Figure 6:**
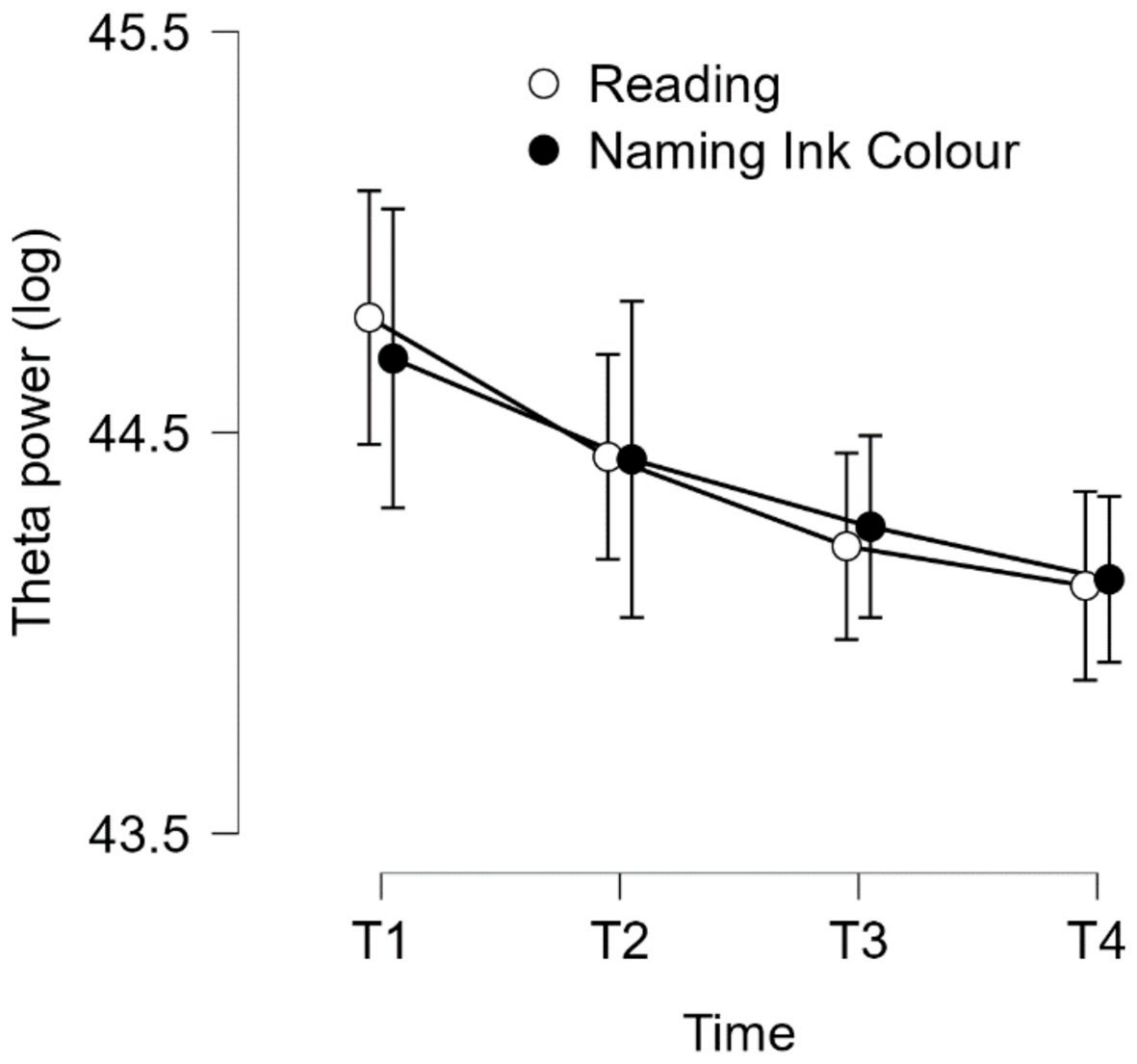
Task-related theta power density as a function of time-on-task (T1-T4) during the modified Stroop task in electrode FP1. Error bars represent standard errors.

Then, to assess the effect of time-on-task on the stimulus-locked theta power density recorded during the modified Stroop task, we performed a repeated-measures ANOVA with Time-on-task (T1, T2, T3 & T4) and Type of trial (reading vs. naming ink color) as within-subjects factors on the 64 electrodes. The results showed that the effect of Time-on-task on the stimulus-locked theta power density reached significance only for 4 electrodes: FP1, P2, CP2, P4. The effect is identical for the 4 electrodes: stimulus-locked theta power decreased with time-on-task. We reported hereafter only the results of the ANOVA for FP1: *F* (2.684, 40.258) = 11.008, *p* < 0.001, *η^2^_p_* = 0.423 (see Figure 6). The effect of Type of trial did not reach significance for the 4 electrodes: *F* (1, 15) = 0.004, *p* = 0.952, *η^2^_p_* = 0.000. In addition, we conducted a repeated-measures MANOVA with Electrodes (F3, Fz and F4; ROI corresponding to the DLFC) with electrodes (F3, Fz and F4), time (T1, T2, T3 and T4) and trial type (naming, reading) as within-subjects factors on stimulus-locked theta spectral density during the Stroop task. Neither the main effect of time nor any of the interactions involving the time factor reached significance. Results concerning the source localization analysis are presented in Supplementary Material S4 and Supplementary Figure S1.

#### 3.1.8 ECG activity during the mental tasks

We conducted 3 separate repeated-measures ANOVAs with Time-on-task (T1, T2, T3 and T4) as the within-subjects factor and Group as the between-subjects factor on the HRV parameters (SDNN, HF & LF). The results for the SDNN index showed a significant interaction of Time-on-task x Group: *F* (2.073, 60.130) = 3.889, *p* = 0.025, *ƞ^2^_p_* = 0.118. This interaction disappeared when conducting ANCOVA with BMI as a covariate: *F* (2.09, 58.62) = 2.198; *p* = 0.118; *ƞ^2^_p_* = 0.073.

For the HF, the interaction of Time-on-task x Group and the simple effect of Group did not reach significance: *F* (2.746, 79.639) = 0.589, *p* = 0.609, *ƞ^2^_p_* = 0.020 and *F* (1 29) = 0.516, *p* = 0.478, *ƞ^2^_p_* = 0.017, respectively. However, the effect of Time-on-task was significant: *F* (2.746, 79.639) = 2.964, *p* = 0.041, *ƞ^2^_p_* = 0.093. HF increased throughout the mental task, regardless of the task.

**Figure 7:**
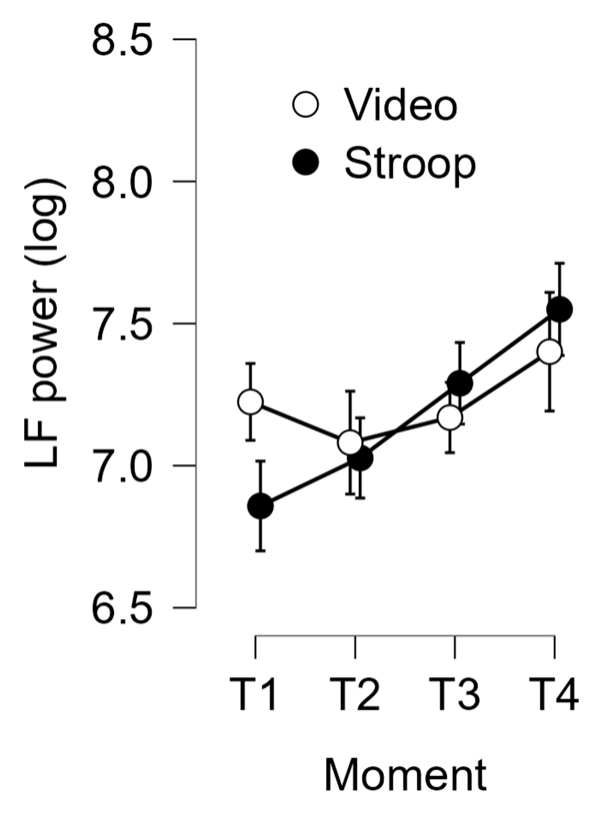
LF power of heart rate variability as a function of time-on-task and group (experimental and control). Error bars represent standard errors.

Finally, concerning the results for LF, the interaction of Time-on-task x Group reached significance: *F* (2.477, 70.959) = 5.104, *p* = 0.005, *ƞ^2^_p_* = 0.150 (see Figure 7). This interaction was still significant when conducting ANOVA with BMI as a covariate: *F* (2.58, 72.24) = 3.40; *p* = 0.028; *ƞ^2^_p_* = 0.108. A breakdown of this interaction showed that LF increased similarly for both groups from T2 to T4, whereas it decreased for the control group between T1 and T2 but increased for the experimental group: *F* (1, 29) = 5.915; *p* = 0.021; *ƞ^2^_p_* = 0.169.

### 3.2 Performance, effort and pain in the TTE handgrip task

#### 3.2.1 Effect of mental fatigue on TTE

**Figure 8:**
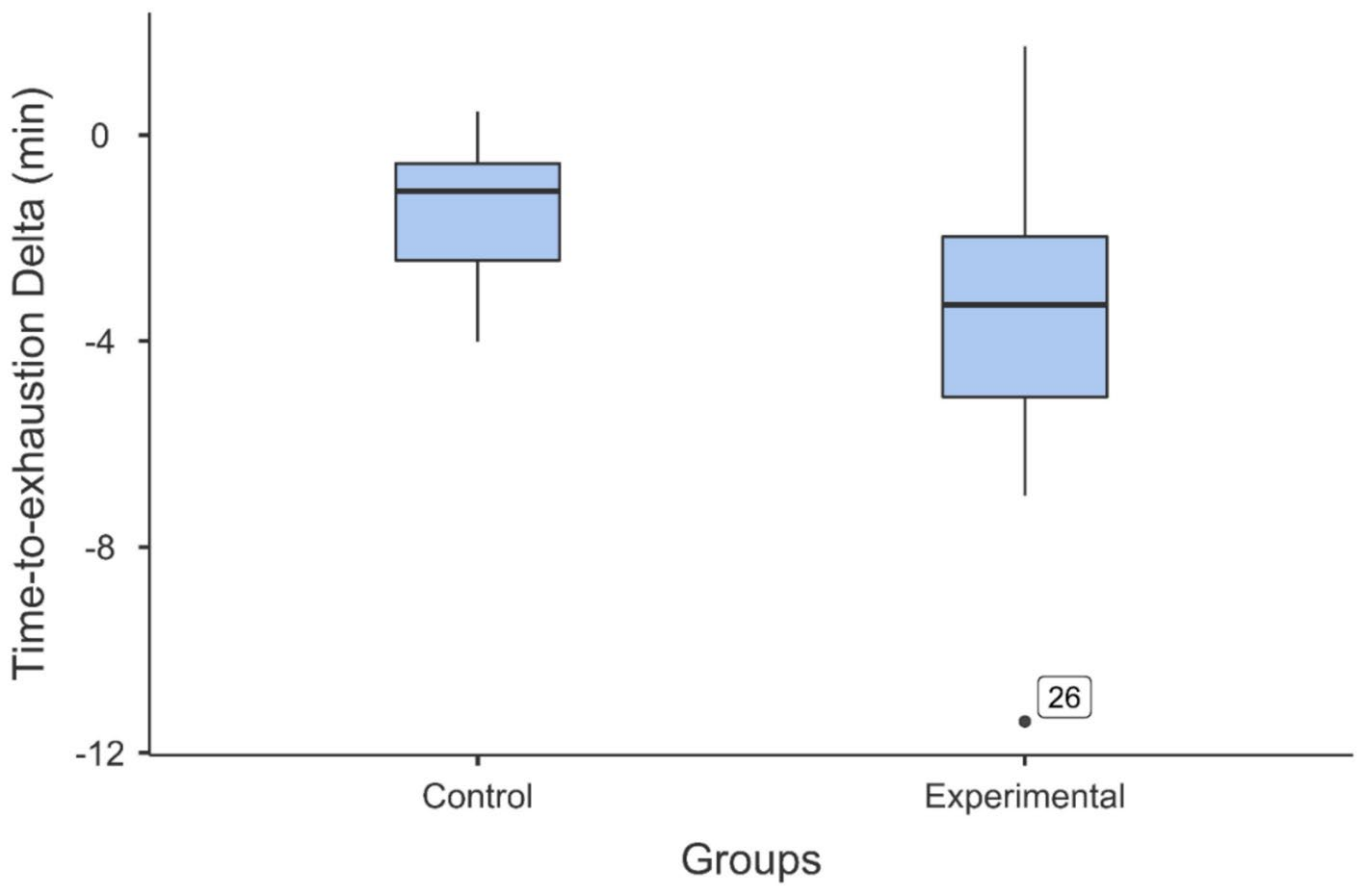
Box and whisker plots illustrating the comparison of the decrease in time-to-exhaustion (time-to-exhaustion delta) between the experimental and control groups. The participants who performed the Stroop task as the fatiguing task (experimental group) showed a larger decrease in performance after this task than the decrease in performance observed in the group of participants who performed the documentary viewing task as the control task (control group). The black dot just above “Experimental” indicates the outlier data of participant 26.

Since the initial performance of the two groups in the handgrip task was not homogeneous, the TTE was calculated separately for each subject as follows: TTE Delta (min) = TTE in the second handgrip task minus TTE in the first handgrip task. The relative decrease was higher in the experimental group (41.5%) than in the control group (27.7%). Finally, to highlight the effect of mental fatigue, the TTE delta of the two groups was compared with the two-sample t test. The results indicated that the TTE delta was significantly lower for the experimental group (*M* = −3.45 min, *SD* = 3.17) than for the control group (*M* = −1.47 min, *SD* = 1.31): *t* (30) = 2.31, *p* = 0.028, *d* = 0.818 (see Figure 8). Complementary analyses are presented in Supplementary Material S5.

A *post hoc* computation of the achieved power, given an alpha of 0.05, a sample size of 16 participants per group, a one-tailed t test and an effect size of 0.818, led to a power value of 1-beta = 0.731.

#### 3.2.2 Perceptions of effort and muscle pain during the TTE handgrip task

**Figure 9:**
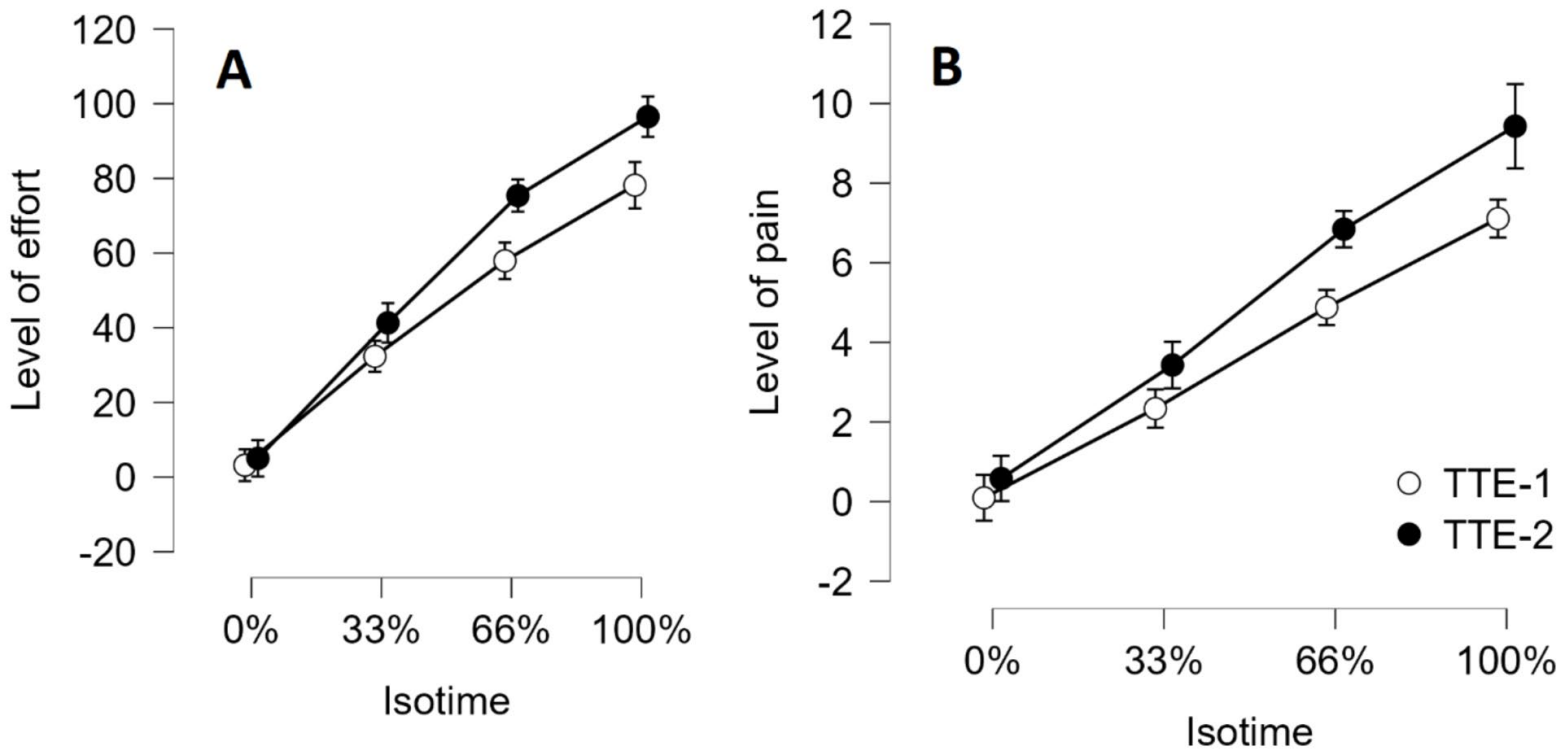
Perception of effort **(A)** and Perception of muscle pain **(B)** during the two TTE handgrip tasks as a function of individual isotime (0, 33, 66, 100%). A significant, linear increase in the effort and pain level as a function of individual isotime was observed. Error bars represent standard errors.

We conducted an ANOVA with Group (Experimental vs. Control) as the between-subjects factor and Time of measurement (before the mental task vs. after the mental task) and Individual isotime (0, 33, 66 and 100%) as within-subjects factors on the perception subjective of effort during the two TTE handgrip tasks. When testing an effect involving individual isotime, we applied a Greenhouse–Geisser correction to consider any violation of the sphericity assumption. The interactions Group x Time of measurement x Individual isotime, Group x Time of measurement and Group x Individual isotime did not reach significance. In contrast, the interaction between Time of measurement and Individual isotime reached significance: *F* (2.11, 63.30) = 11.825, *p* < 0.001, *η^2^_p_* = 0.283 (see Figure 9A). The perception of effort increased more sharply during the second TTE task (TTE-2) than during the first TTE task (TTE-1). The effect of Group was not significant: *F* (1, 30) = 0.321, *p* = 0.575, *η^2^_p_* = 0.011.

The same ANOVA was conducted on the subjective perception of muscle pain during the two TTE handgrip tasks. The interactions Group x Time of measurement x Individual isotime, Group x Time of measurement and Group x Individual isotime did not reach significance. In contrast, the interaction between Time of measurement and Individual isotime reached significance: *F* (1.696, 50.8869) = 6.302, *p* = 0.005, *η^2^_p_* = 0.174 (see Figure 9B). The perception of pain increased more sharply during the second TTE task. Finally, the effect of Group was not significant: *F* (1, 30) = 1.54, *p* = 0.224, *η^2^_p_* = 0.049.

## 4 Discussion

The principal aim of this study was to show an effect of mental fatigue on endurance performance while measuring for effort engagement in the fatiguing and control tasks by employing psychophysiological measurements, such as electrophysiological changes in mid-frontal theta and cardiac reactivity. For that purpose, we used a protocol that already successfully induced an effect of mental fatigue on endurance performance (Mangin et al., 2021; Lorcery et al., 2024). This protocol included a long and effortful fatiguing task (i.e., a modified computerized incongruent Stroop task), a nonboring control task (i.e., a documentary viewing task) and an effortful physically dependent task (i.e., isolation time-to-exhaustion handgrip task at 13% of MVC).

Overall, the participants in each group followed the instructions during the mental tasks. On the one hand, the participants who performed the Stroop task had a percentage of errors less than 12% for the ‘reading’ trials and 20% for the ‘naming ink color’ trials throughout the cognitively demanding task. On the other hand, the participants who performed the documentary viewing task had a percentage of correct responses significantly greater than the chance level. In addition, the participants’ MVC significantly decreased after the TTE handgrip task compared to beforehand, regardless of the group and the moment of the session (before or after the mental task). This decrease in MVC reflects muscular fatigue and suggests that the participants truly contracted their forearm muscles until exhaustion. This inference is confirmed by the significant increase in the perception of pain and effort throughout the TTE handgrip task.

Furthermore, the subjective data showed that the cognitive task performed by the experimental group was considered more difficult than that performed by the control group. However, contrary to our expectations, subjective fatigue increased in both groups throughout the session and did not allow us to distinguish the two tasks. Although some studies have successfully used VAS to distinguish between mentally demanding tasks and the control task (e.g., Boksem et al., 2005; Marcora et al., 2009; Van Cutsem et al., 2017a, 2017b), our results suggest subjective assessment may not always reliably capture mental fatigue. This supports the claim of Pessiglione et al. (2025) that self-report methods suffer from several limitations that mitigate their validity. It seems that in some cases participants confounded fatigue with drowsiness. In the present experiment, the EEG setup required a long preparation process that may have led to a decrease in vigilance from the beginning of the session in both groups, which could explain the lack of significant difference between the two groups. This point needs to be checked in future experiments.

As expected, the mentally demanding Stroop task, which requires repeated inhibitory control and cognitive flexibility, led to a worse performance in the subsequent dependent task compared to the control task. The results concerning overall mid-frontal theta confirmed that the Stroop task required more mental effort than the documentary viewing task in the frontal brain regions. We observed higher theta waveband power mainly in the frontal, prefrontal and central areas during the Stroop task. These results were in accordance with those observed in previous studies as an index of mental effort investment during other mentally demanding tasks (Fairclough and Ewing, 2017). The results of our source localization also showed that the sources of cerebral activation originated mainly from the prefrontal, frontal and central areas and, more precisely, regions close to the anterior cingulate cortex (ACC), thalamus and posterior cingulate cortex (PCC). The two first brain structures were often associated with effort deployment (André et al., 2019).

However, contrary to our expectations, but in agreement with the results observed by Arnau et al. (2021), the results of time-on-task assessment of task-related theta power showed a decrease in theta power throughout the Stroop task, and regardless the type of trial (reading vs. naming ink color), suggesting a progressive disengagement of effort. These results differ from those observed by Van Cutsem et al. (2017a), who reported an increase in overall theta spectral power density during a 45-min modified Stroop task, and from those of Van Cutsem et al. (2018), who found no effect of time on the task during a 90-min modified Stroop task. The difference in results can be easily explained by the fact that Van Cutsem et al. (2017a, 2018) analyzed overall theta as a function of time on the task, whereas we analyzed event-related theta. Arnau et al. (2021) clearly showed, during a 180 min Simon task, that task-unrelated theta varies in the opposite direction to task-related theta. The theta waves recorded by Van Cutsem et al. (2017a, 2018) were actually a mixture of task-related and task-unrelated theta, which explains the divergence in results between our study and theirs. For future studies on mental fatigue, based on Arnau and collaborators’ work, it is recommended to use stimulus-locked task-related theta to measure mental effort engagement over time during the fatiguing task.

In accordance with the EEG results, we also noted a gradual increase in HRV parameters (SDNN, HF & LF) toward the end of the task for the participants who performed the Stroop task. This observation suggested that parasympathetic activity was more dominant toward the end of the Stroop task, indicating that the participants were becoming less and less stressed by the task (habituation process) or were gradually disengaging from the task. These findings closely align with those reported in Van Cutsem et al. (2022) and Lorcery et al. (2024). Future studies could help determine which explanation is most plausible by manipulating the stressful nature of the fatiguing task while keeping mental load constant. For example, this could involve comparing a continuous 30-min Stroop task with a sequence of three 10-min tasks of similar difficulty (e.g., Stroop, Eriksen, Simon).

As a first limitation of this study, we note the small sample size that was not determined *a priori* by a sample size calculation. Although, as mentioned earlier, we have successfully observed the effect of mental fatigue on endurance performance, it is difficult to generalize the results with our limited sample size. However, the results confirmed those already obtained with a similar protocol (Mangin et al., 2021; Lorcery et al., 2024). In addition, the effect size observed in the present study (*d* = 0.818) is very close to the effect size reported in the meta-analysis of Giboin and Wolff (2019) for this type of isolation task (*d* = 0.719). Finally, a post-hoc analysis showed that the achieved power was quite high (1-beta = 0.731).

The dissimilarity between the experimental and control tasks can be mentioned as the second limitation of this study. In the documentary viewing task, the participants were more passive and confronted with a large variety of visual stimuli that did not allow for the measurement of task-related stimulus-locked theta power density. In contrast, in the depleting Stroop task, they were more active (i.e., naming and reading aloud) and confronted with the repetition of a small number of different visual stimuli. However, it was shown in a previous study (Mangin et al., 2021) that the video task was an effective control task because it was not boring but was less effortful than the Stroop task. For instance, it has been shown that the congruent version of the Stroop task is highly boring and, in this way, requires effortful control to continue the task. Finding a control task as similar as possible to the depleting task but requiring little effort and inducing little boredom is challenging. In this way, allowing participants to choose which documentary to watch could further reduce the perceived boringness of the control task.

A third limitation can be raised regarding the use of a small muscle submaximal task as a model of endurance performance. Even if the choice of the isolation TTE handgrip task was guided by the higher sensitivity of this type of task to mental fatigue (Giboin and Wolff, 2019), it could be mentioned that sport situations generally involve whole-body endurance tasks rather than isolation endurance tasks. In future studies on this topic, it would be interesting to use whole-body endurance dependent tasks with a higher sample size and a within-subjects design while controlling effort with psychophysiological indices during the depleting and control tasks (e.g., Proost et al., 2024). In addition, it is well established that time-to-exhaustion tests exhibit significantly greater variability than time-trial tests (Laursen et al., 2007), and are therefore potentially less suitable for research on mental fatigue.

## 5 Conclusion

Overall, this study showed that when studying mental fatigue, it is interesting to measure performance and effort engagement during fatiguing and control tasks to verify whether the fatiguing task requires more effort than the control task, and whether the participants stayed engaged throughout the whole fatiguing task. In the present study, although we observed a disengagement of effort during the Stroop task, we still observed an effect of mental fatigue on physical performance. It would be interesting in the future to investigate if participants use different strategies to manage mental fatigue (e.g., deploying compensatory effort to maintain performance vs. sacrificing performance for energy saving).

## Data Availability

The datasets presented in this study can be found in online repositories. The names of the repository/repositories and accession number(s) can be found at: https://osf.io/deyhn/?view_only=7b00aa67e1cc437c8abedd258031b995.
