# Supplementary material for: Mental fatigue impairs endurance performance in a time-to-exhaustion handgrip task: psychophysiological markers of effort engagement dynamics
Source: Front Psychol. 2025 Jul 23;16:1611135. doi: 10.3389/fpsyg.2025.1611135 (PMC12325378; doi:10.3389/fpsyg.2025.1611135)
Supplement: Supplementary file 1 [file Data_Sheet_1.pdf]

Supplementary Material

S1. Inclusion criteria

The participants should not present one or several of these criteria:

- Presence of a functional problem involving the dominant hand

- Color-blindness

- Insufficiently corrected vision to correctly perform the mental tasks

- Presence of language or learning disorders

- History of severe neurological or metabolic pathologies

- History of psychiatric disorders

- Regular use of psychotropic drugs

- Substance addiction

- Engagement in a muscle-strengthening activity for more than 6 months

S2. Questionnaires used to check for inclusion criteria

Participants first completed a sociodemographic questionnaire (height, weight and age) and a hand laterality scale (Oldfield, 1971) to determine their dominant hand. The participants were also tested for color blindness (Ishihara, 1918) to determine whether they were able to perform the Stroop task. Then, the participants answered questions regarding their general state and health. Certain experimental precautions related to any parameters that could have an effect on mental strain and fatigue were considered, such as not consuming alcohol, caffeine, nicotine or psychostimulant substances; avoiding stressful events and/or intense physical activity several hours before the session; and having a sufficient amount of sleep the night before the experiment.

S3. Performance in the documentary watching task

To verify whether the participants paid attention to the content of the documentary, the percentage of their correct responses was compared to the level of chance (i.e., 50%) via the one-sample t test. According to the results, the participants answered significantly better than chance (*M* = 78.1%, *SD* = 9.11%): t (16) = 12.4, p < .001, d = 3.09.

S4. Complementary EEG analysis

We conducted a source localization analysis. A K-means clustering (K = 33) algorithm using EEGLAB Study Statistics was performed only on the components that represented higher theta power during the Stroop task in the same regions, as previously observed based on the results of our power spectral analysis. To identify the best clusters, first, the outlier clusters recognized automatically by EEGLAB were excluded, and then the clusters including sufficient numbers of participants and components (i.e., at least 8 participants representing more than 50% of the data) were considered. From the results of kmean clustering, 6 of 35 clusters were identified and contained more than 50% of the data. As shown in Figure S1, the sources of cerebral activation originated from the prefrontal, frontal and central areas, more precisely regions close to the anterior cingulate cortex (ACC), thalamus and posterior cingulate cortex.

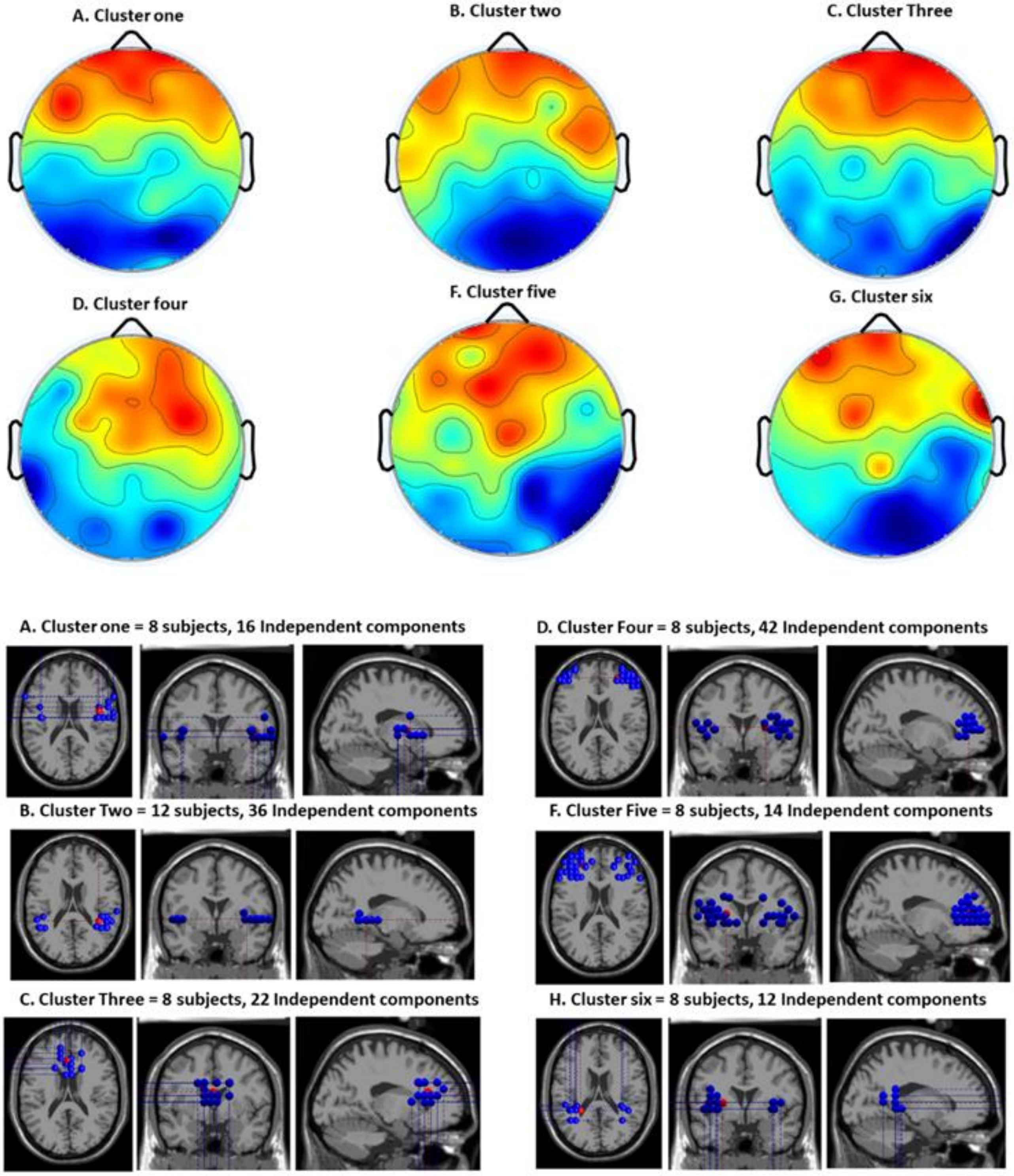

Figure S1: Scalp maps and dipole source locations of the 6 independent component clusters of the 16 participants in the experimental group, which performed the modified Stroop task. The first image on the top represents the scalp maps of each cluster, and the second image on the bottom shows the 3D dipole

source locations and their projections onto the MNI brain template. For each set of three figures illustrating a cluster, there are top, coronal and sagittal views from left to right. The blue dots represent each of the bilaterally symmetric dual dipoles originating from the EEG signal sources, and the red dot is the mean of all these dipoles.

S5. Complementary analyses on the effects of mental fatigue on TTE

The results of the Shapiro-Wilk test for the TTE delta also showed a violation of the assumption of normality: $W = .933$, $p = .046$. Therefore, we used the nonparametric Mann-Whitney U test to avoid any violation of normality, and the results showed a significant difference of Group on TTE: *U* (N Video = 16, N Stroop = 16) = 71, $p = .032$, $r = .455$. The decrease in performance in the experimental group (*M* = 33.5%, *SD* = 30.2%) was larger than the decrease in performance observed in the control group (*M* = 18.2%, *SD* = 25.2%).

Because the observation of the effect of mental fatigue on physical performance is the main objective of this paper, we also conducted an ANCOVA with Group as a between-subjects factor and BMI as a covariate. In this case, the effect of Group no longer reached the significance level but was marginal: F (1, 29) = 3.939, $p = .057$, $\eta^2_p = .120$.

We also performed the same analyses while excluding participants who had a decrease or an increase more than 2 standard deviations from the mean. One participant was removed (point 26 on Figure 8). The results of the t test without the outlier showed a significantly lower TTE delta for the experimental group (*M* = -2.92 min, *SD* = 2.44) compared to the control group (*M* = -1.47 min, SD = 1.31): t (29) = 2.08, $p = .046$, $d = 0.749$. While conducting the ANCOVA with BMI as a covariate and removing the outlier, the effect of Group reached significance: F (1, 28) = 4.464, $p = .044$, $\eta^2_p = .137$.

References

Ishihara, S. (1918). Tests for color blindness. American Journal of Ophthalmology, 1(5), 376.

Oldfield, R. C. (1971). The assessment and analysis of handedness: The Edinburgh inventory. Neuropsychologia, 9(1), 97–113. https://doi.org/10.1016/0028-3932(71)90067-4
